# The profile and prognostic significance of bone marrow T-cell differentiation subsets in adult AML at diagnosis

**DOI:** 10.3389/fimmu.2024.1418792

**Published:** 2024-07-19

**Authors:** Kai Sun, Zong-Yan Shi, Ya-Zhe Wang, Dai-Hong Xie, Yan-Rong Liu, Qian Jiang, Hao Jiang, Xiao-Jun Huang, Ya-Zhen Qin

**Affiliations:** Peking University People’s Hospital, Peking University Institute of Hematology, National Clinical Research Center for Hematologic Disease, Beijing Key Laboratory of Hematopoietic Stem Cell Transplantation, Beijing, China

**Keywords:** AML, memory T cell, naive T cell, *DNMT3A* mutation, prognosis

## Abstract

**Background:**

T lymphocytes in tumor microenvironment play a pivotal role in the anti-tumor immunity, and the memory of T cells contributes to the long-term protection against tumor antigens. Compared to solid tumors, studies focusing on the T-cell differentiation in the acute myeloid leukemia (AML) bone marrow (BM) microenvironment remain limited.

**Patients and methods:**

Fresh BM specimens collected from 103 adult AML patients at diagnosis and 12 healthy donors (HDs) were tested T-cell differentiation subsets by multi-parameter flow cytometry.

**Results:**

CD4 and CD8 T-cell compartments had different constituted profiles of T-cell differentiated subsets, which was similar between AML patients and HDs. Compared to HDs, AML patients as a whole had a significantly higher proportion of CD8 effector T cells (Teff, *P* = 0.048). Moreover, the T-cell compartment of AML patients with no *DNMT3A* mutations skewed toward terminal differentiation at the expense of memory T cells (CD4 Teff: *P* = 0.034; CD8 Teff: *P* = 0.030; CD8 memory T: *P* = 0.017), whereas those with mutated *DNMT3A* had a decrease in CD8 naïve T (Tn) and CD4 effector memory T cells (Tem) as well as an increase in CD4 central memory T cells (Tcm) (*P* = 0.037, 0.053 and 0.053). Adverse ELN genetic risk correlated with a lower proportion of CD8 Tn. In addition, the low proportions of CD4 Tem and CD8 Tn independently predicted poorer relapse-free survival (RFS, HR [95%CI]: 5.7 (1.4–22.2), *P* = 0.017 and 4.8 [1.3–17.4], *P* = 0.013) and event-free survival (EFS, HR [95% CI]: 3.3 (1.1–9.5), *P* = 0.029; 4.0 (1.4–11.5), *P* = 0.010), respectively.

**Conclusions:**

AML patients had abnormal profiles of BM T-cell differentiation subsets at diagnosis, which was related to *DNMT3A* mutations. The low proportions of CD4 Tem and CD8 Tn predicted poor outcomes.

## Introduction

Acute myeloid leukemia (AML) is an aggressive hematological malignancy characterized by an accumulation of immature cells of the myeloid lineage. It is not just leukemic cells themselves that give rise to the disease, but AML patients usually have abnormal profiles of the bone marrow (BM) microenvironment, which also makes an indispensable contribution to the pathogenesis of AML (1).

T lymphocytes in tumor microenvironment play a central role in the anti-tumor immunity. Memory of T cells is a key mechanism for the long-term protection against diverse pathogens, including tumor antigens (2). To date, many studies have reported the population changes and prognostic significance of tumor-infiltrating and circulating T cells grouped by differentiation sub-populations in various solid tumors (3–6).Compared to the solid tumors, researches focusing on the profile of BM T-cell differentiation subsets in AML remain limited. Xu et al. investigated 10 newly diagnosed AML cases and supposed that memory T cells skewed toward terminal differentiation in the CD8 T-cell population in AML patients compared with healthy individuals (7). Schnorfeil et al. reported that T-cell compartment shifted toward the effector memory phenotype in relapsed AML patients compared to diagnosis (8). Furthermore, the prognostic role of BM T-cell differentiation subsets in AML needs to be clarified.

The widely accepted T-cell differentiation model is that memory T cells are generated from effector T cells through epigenetic modifications, and DNA methyltransferase 3A (*DNMT3A*) is the critical regulator of effector versus memory fate decisions (9, 10). Notably, *DNMT3A* mutations commonly occur in AML with an incidence of around 20%, and are recognized as clonal hematopoiesis related mutations (11). It was reported that *DNMT3A* mutations were detectable in T and B lymphocytes except for leukemic cells in a certain number of AML cases (12). However, it remains unknown whether *DNMT3A* mutations in AML influence the differentiation of T cells.

In the present study, by performing multi-parameter flow cytometry (MFC) using fresh BM samples collected from AML patients at diagnosis, we established the profile of T-cell differentiation subsets and explored their prognostic significance.

## Material and methods

### Patients and treatment

A total of 103 newly diagnosed adult non-M3 AML cases and 12 healthy donors (HDs) who were aspirated BM specimens in our institute from February 2022 to March 2023 were included in the present study. The median age of all patients was 48 (range 16–64) years at diagnosis, and sixty-one (59.2%) patients were male. The diagnosis was based on bone marrow morphology, immuno-phenotyping, karyotyping and molecular biology. Patients’ baseline clinical characteristics were summarized in **Table 1**. As we previously reported, all patients were screened AML-related fusion transcripts (*RUNX1::RUNX1T1*, *PML::RARA*, *CBFB::MYH11*, *DEK::NUP214*, *BCR::ABL1* and *KMT2A* rearrangements) as well as *FLT3-ITD* and *NPM1* mutations (13, 14). 93, 72 and 2 patients were individually tested *TP53*, *CEBPA* and *DNMT3A* mutations using Sanger sequencing (15, 16). In addition, 70 patients underwent targeted next-generation sequencing for screening AML-related gene mutations.

**Table 1:** Patients’ clinical characteristics at diagnosis.

Variable	Number of patients or median (range)
All	103
Age (y)	48 (16–64)
Male (%)	61 (59.2%)
WBC count (×10^9^/L)	16.6 (1.2–460.0)
Hemoglobin (g/L)	87 (36–152)
Platelet count (× 10^9^/L)	40 (4–507)
BM blast (%)	61 (22–98)
FAB subtypes	
M1	4 (3.9%)
M2	73 (70.9%)
M4	22 (21.4%)
M5	3 (2.9%)
M7	1 (1.0%)
2022-ELN genetic risk classification (n = 89) *	
Favorable	38 (42.7%)
Intermediate	25 (28.1%)
Adverse	26 (29.2%)

The symbol * means the ELN risk classification by genetics were defined based on the 2022 ELN guidelines.

Overall, 79 (76.7%) patients received treatment and were followed up at our institute. As previously reported, induction regimen involved IA (idarubicin and cytarabine), HAA (homoharringtonine, aclarubicin and cytarabine), AA (aclarubicin and cytarabine) or CAG (cytarabine, aclarubicin and G-CSF), and those unfitted for intense chemotherapy received azacitidine combined with targeted therapy (Dasatinib, Sorafenib or Venetoclax). The consolidation therapy included chemotherapy alone or chemotherapy followed by allogeneic hematopoietic stem-cell transplantation (allo-HSCT). The indications for allo-HSCT, conditioning regimen and graft-versus-host disease prophylaxis were comprehensively described previously (17). The cutoff date for the last follow-up was October 2023.

This research was approved by the Ethics Committee of the Peking University People’s Hospital and was in accordance with the Declaration of Helsinki.

### Sample preparation for flow cytometry analysis

Fresh BM specimens collected from 103 AML patients at diagnosis and 12 HDs were tested T-cell differentiation sub-populations by MFC. First, phosphate buffered saline (PBS) was used to wash samples for three times, and directly-conjugated monoclonal antibodies were then incubated for 15 min in the dark at room temperature. Subsequently, FACS lysis solution (BD Biosciences, San Jose CA, USA) was applied to lysing red blood cells for 10 min. After lysis, cells were washed, resuspended with PBS and kept at 4°C until acquisition. FACSCanto™ II (BD Biosciences, San Jose CA, USA) and Navios (Beckman Coulter Life Sciences, Indianapolis IN, USA) were used for data collection, and Kaluza 2.0 (Beckman Coulter, Brea, CA, USA) was used for data analysis.

### Antibody panel

The antibody panel for the T-cell differentiation sub-population testing included CD45-V500 (BD Biosciences, Clone HI30), CD3-APC-H7 (BD Biosciences, Clone SK7), CD4-Alexa Fluor (Biolegend, Clone OKT4), CD8-PE-Cy7 (BD Biosciences, Clone SK1), CCR7-FITC (Biolegend, Clone G043H7), CD45RO-PerCP (Biolegend, Clone UCHL1), and CD95-BV421 (Biolegend, Clone DX2).

### Gating strategy

**Figure 1:**
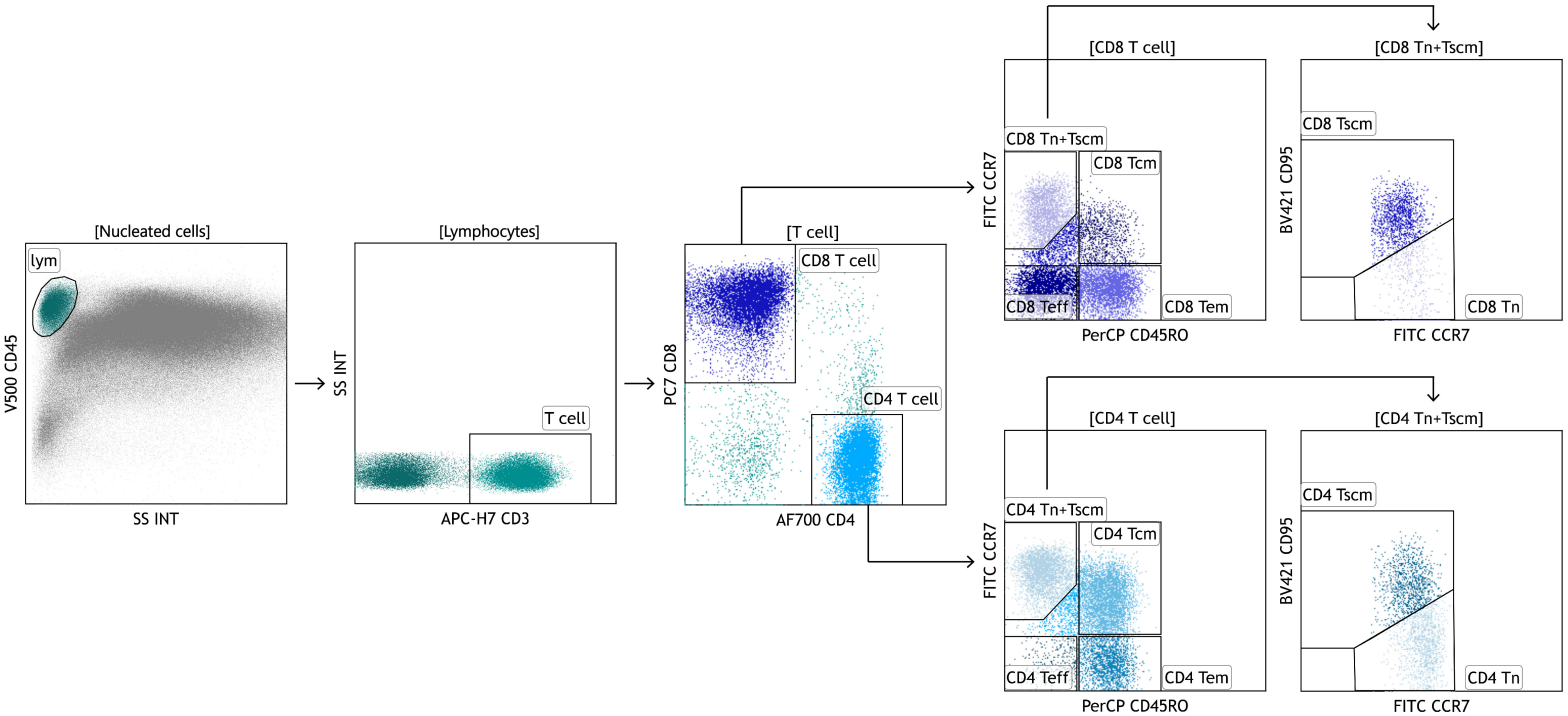
The gating strategy of CD4 and CD8 T-cell differentiation subsets tested by multi-parameter flow cytometry (MFC).

The gating strategy of T-cell sub-populations referred to what Lugli et al. reported (18) and was shown in **Figure 1**. The naïve T cells (Tn), stem central memory T cells (Tscm), central memory T cells (Tcm), effector memory T cells (Tem), and terminally differentiated effector T cells (Teff) were individually defined as CD45RO^-^CCR7^+^CD95^-^, CD45RO^-^CCR7^+^CD95^+^, CD45RO^+^CCR7^+^, CD45RO^+^CCR7^-^ and CD45RO^-^CCR7^-^, and their proportions represented their percentages in CD4 or CD8 T cells.

### Definitions and statistical analysis

Relapse-free survival (RFS) and event-free survival (EFS) were two endpoints for patients’ follow-up in the present study. Complete remission (CR) referred to morphologic CR (19), and RFS was measured from CR to relapse, or to the last date of BM morphology examination. EFS was measured from the date of diagnosis to not achieving CR after two courses of induction or death from any cause, or from CR to relapse (20).

*Mann–Whitney* U or *Kruskal–Wallis* test was performed for the pairwise comparisons of continuous variables. *Fisher’s* exact test was performed for the comparisons of categorical variables. Survival functions were estimated using Kaplan–Meier method and compared using log-rank test. Variables associated with *P* < 0.20 in univariate analysis were entered into Cox model-based multivariable analysis. *P* values less than 0.05 were considered statistically significant. SPSS 26.0 software package (SPSS Inc., Chicago, IL, USA), GraphPad Prism 9 (GraphPad Software Inc., La Jolla, CA, USA) and R 4.2.3 (R Foundation for Statistical Computing, Vienna, Austria) were used for data analysis.

## Results

### Patient outcomes

Of all 103 AML patients included, 79 (76.7%) patients received treatment and were followed up for a median period of 8.6 (0.6–19.5) months. 55 (69.6%), 17 (21.5%) and 3 (3.8%) patients individually achieved CR after 1, 2 and 3 cycles of induction chemotherapy, 3 patients (3.8%) did not achieve CR after >3 cycles of induction, and 1 (1.3%) died before CR achievement. Among 75 (92.6%) patients who achieved CR, 53 (70.7%) patients received chemotherapy alone as consolidation chemotherapy and were designated as the chemotherapy group, 15 (27.8%) of whom experienced subsequent relapse; the remaining 22 (29.3%) patients received chemotherapy followed by allo-HSCT at the first CR (matched sibling donor, n = 1; haploidentical related donor, n = 20; matched unrelated donor, n = 1), and 2 relapsed after transplantation. The 2-year RFS and EFS rates were 75.4% (95% confidence interval [CI] 61.2–84.9%) and 66.6% (95% CI: 52.7–77.3%), respectively.

### The distribution patterns of BM T-cell differentiation subsets of AML and HDs

**Figure 2:**
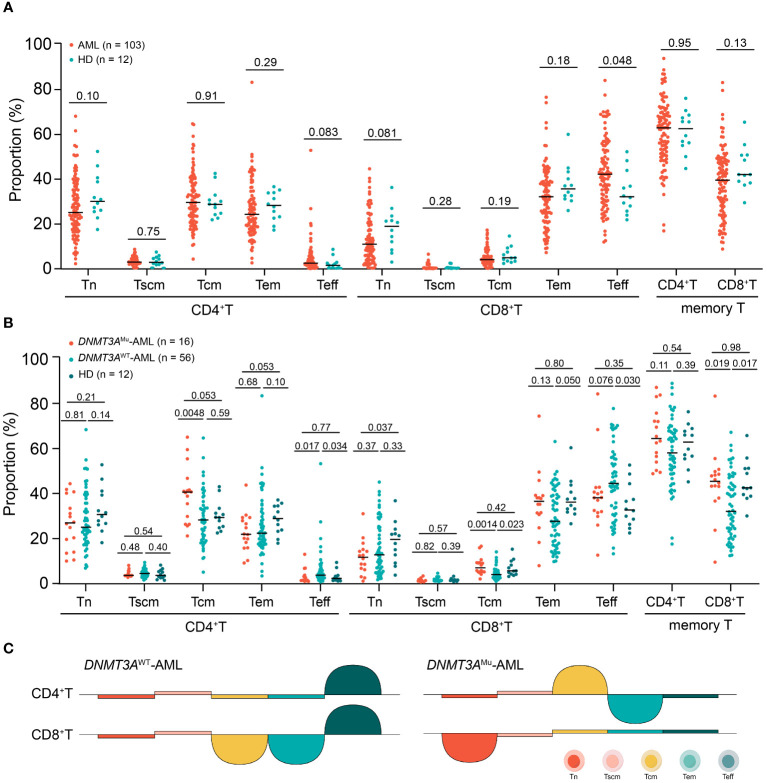
The comparisons of each CD4 and CD8 T-cell differentiation subset between AML patients and HDs **(A)**, among *DNMT3A* ^Mu^-AML, *DNMT3A* ^WT^-AML patients and HDs (Values above the horizontal line represent the *P* values **(B)**, the summary chart of differences of comparisons (Half circle upwards represents higher, downwards represents lower **(C)**.

The distributions of CD4 and CD8 T-cell differentiation subsets of the individual AML patients and HDs were shown in **Supplementary Figure 1** and the comparisons between patients and HDs were shown in **Figure 2A** and **Supplementary Table 1**. The T-cell differentiated profiles were distinct between CD4 and CD8 T-cell compartments in AML patients, which was similar in HDs; That is, Tn, Tcm and Tem accounted for the majority of CD4 T cells, all of which were significantly higher than the proportions of CD4 Tscm and Teff; whereas Tem and Teff were the major sub-populations of CD8 T cells, and both were individually significantly higher than the proportions of CD8 Tn, Tscm and Tcm (all *P* < 0.0010). However, the distribution of T-cell differentiation subsets in AML patients was different from HDs. Compared to HDs, AML patients tended to have a decreased proportion of CD4 Tn (median (range) 25.3% (2.5%-68.2%) vs 30.3% (17.7%-52.5%), *P* = 0.10), as well as an increased proportion of CD4 Teff (2.7% (0.2%-53.0%) vs 1.7% (0.5%-8.9%), *P* = 0.083). Notably, similar to the CD4 T-cell compartment, AML patients had significantly higher CD8 Teff proportion than HDs, whereas the CD8 Tn proportion tended to be lower (Teff, 42.3% (12.1%-84.1%) vs 32.3% (22.0%-52.4%), *P* = 0.048; Tn, 11.2% (0.6%-44.7%) vs 19.1% (3.2%-36.5%), *P* = 0.081). The proportions of the sum of memory T cells and all memory subsets including Tscm, Tcm and Tem of both CD4 and CD8 T-cell compartments were similar between AML patients and HDs (all *P* > 0.10).

### The distribution of T-cell differentiation sub-population correlates with patient age and ELN risk classification

The associations of the proportion of T-cell differentiation sub-populations with patients’ baseline clinical characteristics were analyzed. Correlations existed between the distribution of T-cell differentiation sub-populations and patient age (**Supplementary Table 2**, **Supplementary Figure 2**). In the CD4 T-cell population, the proportions of Tn and Tscm cells individually negatively correlated with age (*P* = 0.031 and 0.022), and that of Tcm positively correlated with age (*P* = 0.0061). Similarly, in CD8 T-cell population, the proportions of Tn and Tscm individually showed and tended to show significant negative correlations with age (*P* < 0.0010 and = 0.085), and the proportions of both Tcm and Tem showed positive correlations with patient age (*P* = 0.0022 and 0.030), respectively. As shown in **Supplementary Figure 2**, HDs had the same trend with AML patients except for the sub-populations of CD4 and CD8 Teff though the statistical differences were not significant, partially because of the small sample size. Moreover, the age of AML patients enrolled was similar to that of HDs (48 (16–64) vs 48.5 (24–65), *P* = 0.49). This excluded the influence of age on the comparison of T-cell differentiation subsets between AML and HDs.

The proportion of Tn was associated with ELN genetic risk classification in AML patients. Patients with adverse ELN genetic risk had significantly decreased proportion of CD8 Tn than those with intermediate risk (6.3% (0.6%-36.4%) vs 13.9% (2.0%-39.1%), *P* = 0.0070), and tended to have decreased CD8 Tn proportion than those with favorable risk (6.3% (0.6%-36.4%) vs 10.4% (1.1%-44.7%), *P* = 0.093). The tendency was similar for the proportion of CD4 Tn sub-population (ELN adverse risk vs intermediate risk: 22.7% (10.0%-54.6%) vs 30.8% (9.5%-55.2%), *P* = 0.054).

Other parameters including gender, WBC count, hemoglobin content, platelet count, and the percentage of BM blast at diagnosis were not related to the proportions of T-cell differentiation subsets (all *P* > 0.05).

### *DNMT3A* mutation correlates with the distribution of T-cell differentiation subsets in AML

Totally 16 out of 72 patients (22.2%) who were tested *DNMT3A* mutations had mutated *DNMT3A*. Compared to those without mutated *DNMT3A* (*DNMT3A* ^WT^), patients with *DNMT3A* mutations (*DNMT3A* ^Mu^) had or tended to have a significantly lower Teff and higher memory T-cell proportions in both CD4 and CD8 T cells (median (range): CD4 Teff: 1.2% (0.4%-12.6%) vs 3.2% (0.2%-53.0%), *P* = 0.017; CD8 Teff: 37.8% (12.1%-84.1%) vs 44.1% (12.7%-77.6%), *P* = 0.076; CD4 memory T: 64.1% (48.5%-87.0%) vs 57.8% (17.1%-88.3%), *P* = 0.11; CD8 memory T: 45.1% (9.0%-83.1%) vs 31.7% (11.9%-66.9%); *P* = 0.019, **Figure 2B**). Within memory T-cell subsets, *DNMT3A* ^Mu^ patients had a significantly higher proportion of Tcm than *DNMT3A* ^WT^ patients (CD4 Tcm: 40.3% [20.8%-64.8%] vs 27.9% [4.5%-64.4%], *P* = 0.0048; CD8 Tcm: 6.4% [1.5%-16.1%] vs 3.4% [0.5%-13.6%], *P* = 0.0014, **Figure 2B**). Therefore, *DNMT3A* mutations in AML are related to the shift of T-cell compartments from the effector to the memory stage.

We further individually compared *DNMT3A* ^Mu^ and *DNMT3A* ^WT^-AML patients with HDs (**Figure 2B**). *DNMT3A* ^WT^ patients had significantly higher CD4 and CD8 Teff proportions and lower CD8 memory T cells than HDs (*P* = 0.034, 0.030 and 0.017), respectively. Within memory T cells, *DNMT3A* ^WT^ patients had a significantly lower CD8 Tcm proportion (*P* = 0.023), and tended to have lower CD4 and CD8 Tem proportions than HDs (*P* = 0.10 and 0.050), respectively. As for *DNMT3A* ^Mu^-AML patients, they displayed similar Teff and memory T cell proportions to HDs (all *P* ≥ 0.35), but a significantly decreased CD8 Tn than HDs (*P* = 0.037); within memory T cells, *DNMT3A* ^Mu^-AML patients displayed a tendency of increased Tcm and decreased Tem in CD4 cells compared with HDs (both *P* = 0.053), respectively. To this extent, compared to HDs, *DNMT3A* ^WT^-AML patients showed a significant T-cell compartment shift from the memory phenotype to Teff, while *DNMT3A* ^Mu^-AML patients had a decreased proportion in CD8 Tn and an inverse distribution of Tcm and Tem within CD4 memory T cells. The summary chart of the comparison results was summarized in **Figure 2C**.

### Low CD4 Tem and CD8 Tn proportions independently predict relapse

**Figure 3:**
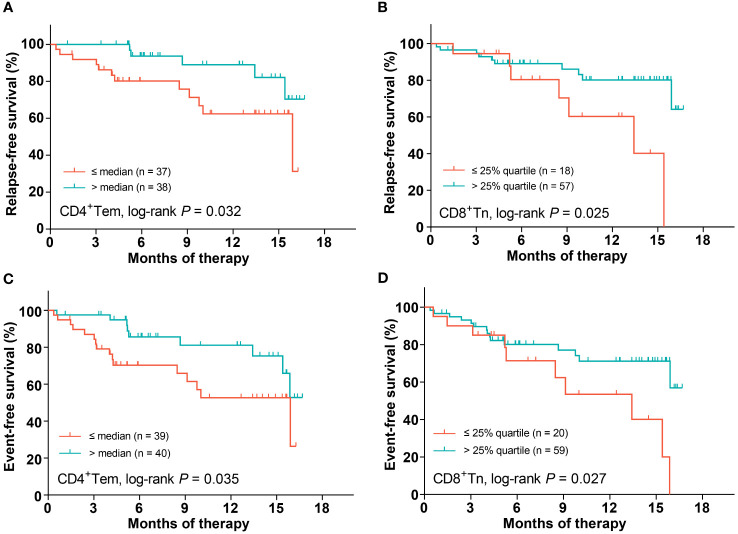
The impact of the proportions of CD4 Tem and CD8 Tn on patients’ survival. CD4 Tem on RFS **(A)**, CD8 Tn on RFS **(B)**, CD4 Tem on EFS **(C)**, CD8 Tn on EFS **(D)**.

Firstly, we quartered patients based on the proportion of each T-cell differentiation sub-population to evaluate its impact on relapse (**Supplementary Figures 3A–J**). Only the proportions of CD4 Tem and CD8 Tn showed a significant tendency (both *P* < 0.20). Then, the optimal cutoff values for the proportion of CD4 Tem and CD8 Tn were individually determined as the median (value: 22.8%) and the 25% quartile (value: 6.0%) according to the trends of survival function curves (**Supplementary Figures 3D, F**). As a result, low proportions of CD4 Tem and CD8 Tn were significantly related to poor RFS (2-year RFS rates: CD4 Tem: 62.4% [95% CI: 41.3%-77.7%] vs 89.0% [68.9%-96.4%], *P* = 0.032; CD8 Tn: 60.3% [27.6%-82.0%] vs 80.1% [64.5%-89.4%], *P* = 0.025, **Table 2**, **Figures 3A, B**), respectively. Among other parameters, only chemotherapy alone was significantly related to a lower RFS rate compared with allo-HSCT (2-year RFS rate: 66.4% [48.0%-79.5%] vs 93.3% [61.3%-99.0%], *P* = 0.016, **Table 2**). Multivariate analysis showed that low proportions of CD4 Tem and CD8 Tn, ELN-intermediate risk (ELN-favorable risk as reference), and chemotherapy alone were independent poor prognostic factors for RFS (hazard ratio (HR): 5.7 (95% CI: 1.4–22.2), *P* = 0.013; 4.8 (1.3–17.4), *P* = 0.017; 6.0 (1.4–25.6), *P* = 0.016; 11.5 (2.1–64.1), *P* = 0.0053, **Table 2**), respectively.

**Table 2:** Univariate analysis and multivariate analysis of RFS (n = 75).

	Univariate analysis		Multivariate analysis	
Variables	2-year RFS (95% CI)	*P* value	HR (95% CI)	*P* value
Tem/CD4^+^T		**0.032**		**0.017**
≤ median (n = 37)	62.4% (41.3%-77.7%)		5.7 (1.4–22.2)	
> median (n = 38)	89.0% (68.9%-96.4%)		1.0	
Tn/CD8^+^T		**0.025**		**0.013**
≤ 25% quartile (n = 18)	60.3% (27.6%-82.0%)		4.8 (1.3–17.4)	
> lower quartile (n = 57)	80.1% (64.5%-89.4%)		1.0	
Age (y)		0.76		
15–45 (n = 38)	77.1% (56.5%-88.8%)			
46–65 (n = 37)	73.5% (51.4%-86.7%)			
Gender		0.24		
Male (n = 41)	72.3% (52.9%-84.7%)			
Female (n = 34)	78.9% (54.2%-91.3%)			
WBC count (× 10^9^/L)		0.54		
≤ 20 (n = 43)	77.0% (54.2%-89.4%)			
> 20 (n = 32)	71.9% (51.2%-85.0%)			
Hemoglobin (g/L)		**0.13**		0.20
≤ 90 (n = 42)	68.7% (50.1%-81.6%)		–	
> 90 (n = 33)	87.0% (62.2%-96.0%)		–	
Platelet count (× 10^9^/L)		0.44		
≤ 40 (n = 37)	80.5% (60.9%-91.0%)			
> 40 (n = 38)	71.1% (49.4%-84.8%)			
BM blast (%)		0.38		
≤ 60 (n = 40)	78.8% (58.0%-90.1%)			
> 60 (n = 35)	71.3% (49.4%-85.0%)			
ELN risk category by genetics* (n = 69)		**0.25**		**0.023**
Favorable (n = 32)	87.2% (64.4%-95.8%)	–	1.0	–
Intermediate (n = 17)	69.6% (37.1%-87.6%)	0.24	6.0 (1.4–25.6)	**0.016**
Adverse (n = 20)	56.5% (19.7%-81.8%)	0.14	–	0.054
*DNMT3A* mutations (n = 63)		0.50		
No (n = 51)	79.8% (62.5%-89.7%)			
Yes (n = 12)	61.1% (19.5%-86.2%)			
Induction therapy (n = 75)		0.46		
IA/HAA (n = 55)	80.0% (64.4%-89.3%)			
AA/CAG (n = 8)	57.1% (17.2%-83.7%)			
Azacitidine + Venetoclax (n = 11)	60.6% (7.5%-90.8%)			
Others (n = 1)	100%			
CR after 1-course induction (n = 74)		0.36		
No (n = 19)	71.3% (38.8%-88.7%)			
Yes (n = 55)	77.0% (60.6%-87.2%)			
Consolidation therapy (n = 74)		**0.016**		**0.0053**
Chemotherapy alone (n = 52)	66.4% (48.0%-79.5%)		11.5 (2.1–64.1)	
Allo-HSCT (n = 22)	93.3% (61.3%-99.0%)		1.0	

Variables with P value <0.20 in the univariate analysis were entered into multivariable analysis.

*The ELN risk classification by genetics were defined based on the 2022 ELN guidelines (19).

The bold values mean significantly different or included in multivariate analysis.

### Low CD4 Tem and CD8 Tn proportions independently predict poor EFS

Similar to the analysis of RFS, the median proportion of CD4 Tem as well as the lower quartile proportion of CD8 Tn was individually used as the cutoff value in the light of the trends of corresponding EFS curves (**Supplementary Figures 3N, P**). Lower proportions of CD4 Tem and CD8 Tn were significantly related to lower EFS rates (2-year EFS rates: CD4^+^Tem: 52.7% [95% CI: 32.9%-69.1%] vs 81.1% [61.9%-91.3%], *P* = 0.035; CD8^+^Tn: 53.5% [24.7%-75.6%] vs 71.1% [55.4%-82.2%], *P* = 0.027, **Table 3**, **Figures 3C, D**), respectively. In addition, patients with ELN-adverse risk had a significantly lower EFS rate than those with favorable ELN risk (2-year EFS rate: 87.2% [64.4%-95.8%] vs 40.1% [11.8%-67.7%], *P* = 0.0035, **Table 3**). Multivariate analysis showed that both low proportions of CD4 Tem and CD8 Tn independently predicted poor EFS (HR (95% CI): 3.3 (1.1–9.5), *P* = 0.029; 4.0 (1.4–11.5), *P* = 0.010, **Table 3**). Neither ELN-genetic risk classification nor consolidation therapy modality independently predicted EFS (*P* = 0.10 and 0.22, respectively).

**Table 3:** Univariate analysis and multivariate analysis of EFS (n = 79).

	Univariate analysis		Multivariate analysis	
Variables	2-year EFS (95% CI)	*P* value	HR (95% CI)	*P* value
Tem/CD4^+^T		**0.035**		**0.029**
≤ median (n = 39)	52.7% (32.9%-69.1%)		3.3 (1.1–9.5)	
> median (n = 40)	81.1% (61.9%-91.3%)		1.0	
Tn/CD8^+^T		**0.027**		**0.010**
≤ lower quartile (n = 20)	53.5% (24.7%-75.6%)		4.0 (1.4–11.5)	
> lower quartile (n = 59)	71.1% (55.4%-82.2%)		1.0	
Age (y)		0.70		
15–45 (n = 38)	65.1% (44.5%-79.6%)			
46–65 (n = 41)	67.9% (47.5%-81.8%)			
Gender		**0.061**		0.62
Male (n = 46)	60.3% (42.3%-74.3%)		–	
Female (n = 33)	75.1% (50.3%-88.7%)		–	
WBC count (× 10^9^/L)		**0.11**		0.11
≤ 20 (n = 44)	73.3% (51.8%-86.3%)		–	
> 20 (n = 35)	57.9% (38.1%-73.3%)		–	
Hemoglobin (g/L)		0.44		
≤ 90 (n = 45)	63.2% (45.2%-76.7%)			
> 90 (n = 34)	73.3% (49.8%-87.1%)			
Platelet count (× 10^9^/L)		0.29		
≤ 40 (n = 37)	74.8% (55.1%-86.8%)			
> 40 (n = 42)	59.5% (39.4%-75.0%)			
BM blast (%)		**0.14**		0.24
≤ 60 (n = 41)	74.7% (54.8%-86.8%)		–	
> 60 (n = 38)	57.4% (36.5%-73.6%)		–	
ELN risk category by genetics* (n = 73)		**0.012**		0.10
Favorable (n = 32)	87.2% (64.4%-95.8%)	–	–	–
Intermediate (n = 19)	58.8% (29.2%-79.6%)	**0.069**	–	0.45
Adverse (n = 22)	40.1% (11.8%-67.7%)	**0.0035**	–	0.080
*DNMT3A* mutations (n = 66)		0.30		
No (n = 53)	72.1% (55.0%-83.6%)			
Yes (n = 13)	43.5% (11.0%-73.1%)			
Induction therapy (n = 79)		0.75		
IA/HAA (n = 56)	70.7% (54.9%-81.9%)			
AA/CAG (n = 9)	57.1% (17.2%-83.7%)			
Azacitidine + Venetoclax (n = 12)	55.0% (8.6%-86.4%)			
Others (n = 2)	50.0% (0.6%-91.0%)			
Consolidation therapy (n = 75)		**0.14**		0.22
Chemotherapy alone (n = 53)	58.9% (39.1%-74.2%)		–	
Allo-HSCT (n = 22)	83.0% (55.3%-94.3%)		–	

Variables with P value <0.20 in the univariate analysis were entered into multivariable analysis.

*The ELN risk classification by genetics were defined based on the 2022 ELN guidelines (19).

The bold values mean significantly different or included in multivariate analysis.

## Discussion

Adoptive cell transfer (ACT) is one of the major immunotherapeutic strategies in AML. Compared to terminally differentiated T cells, donor lymphocyte infusions (DLI) in relapsed AML patients with higher proportions of early-differentiated memory T cells showed superior durability and anti-tumor activity (21). In addition, early-differentiated memory T cells expressed higher levels of immune checkpoints compared relapsed to CR AML patients after allo-HSCT (22). These findings indicated the potential treatment and prognostic significance of exploring T-cell memory sub-populations in AML. In the current study, we used BM rather than peripheral blood specimens, investigating the profile of T-cell differentiation subsets at the primary site of leukemic cells. To our knowledge, this is the first large-scale cohort study concentrating on the profile and prognosis of BM T-cell differentiation subsets at diagnosis in AML.

At first, we established the differentiation subset profile of tumor-infiltrating T cells in patients with newly diagnosed AML. The distribution pattern of T-cell differentiation subsets within both CD4 and CD8 T-cell compartments was similar between AML patients and HDs; but interestingly, CD4 and CD8 populations had constituent differences. Tcm and Tem as well as Tn constituted the major component of CD4 T cells, whereas Tem and Teff accounted for the majority of CD8 T cells. Although not specifically stated, Xu et al. and Noviello et al. showed similar profile distributions to ours in newly diagnosed, CR and relapsed AML patients (7, 22). The mechanism underlying the difference was unclear though, it might be related to the fact that CD8 T-cell population exerted direct killing effects, whereas CD4 T-cell population might mainly function as helper cells.

Xu et al. previously illustrated that Tscm and Tcm frequencies decreased and the frequency of Tem increased compared to HDs by testing 10 AML cases (7). However, we found that none of these frequencies as well as the sum of memory T cells were significantly different between AML patients and HDs based on a large sample size. In contrast, we found that AML patients as a whole had a significantly higher CD8 Teff proportion than HDs, which suggested that leukemia cells existing in AML BM microenvironment might trigger a strong CD8 T-cell response and cause CD8 T cells differentiation to the effector phase. The CD4 T-cell compartment displayed a similar tendency with the higher proportion of Teff and lower proportion of Tn in AML patients. These results indicated the occurrence of a strong T-cell anti-tumor response in AML.

Next, we thought to identify factors that might influence the distribution of T cells in AML differing from HDs. *DNMT3A* controlled the stability of the differentiated state in CD4 T cells, and was critical for restraining the number of memory precursor effector cells and limiting long-term CD8 T-cell memory (9, 10). Mutations of *DNMT3A* caused loss of function through reducing its methyltransferase activity (9, 23). *DNMT3A* is one of the most common mutated genes that drives clonal hematopoiesis. Furthermore, *DNMT3A* mutation highly occurs in AML patients (24, 25). It has been demonstrated that *DNMT3A* mutation arises early in AML evolution, probably in pre-leukemic hematopoietic stem cells (HSCs) from which AML evolves and leads its clonal expansion (11, 26). Therefore, mutated *DNMT3A* exists not only in leukemic cells but also in non-leukemic compartments such as lymphocytes and the functionally normal HSCs in AML (27, 28). T cells had detectable *DNMT3A* mutations were individually reported in 70.5% (12/17) and 100% (2/2) of *DNMT3A*-mutated AML patients (12, 29). In the current study, we had demonstrated that *DNMT3A* mutation in AML was associated with a higher proportion of memory T cells and a lower proportion of Teff.

Since *DNMT3A* mutation had an opposite influence on the phenomenon of increased proportion of Teff in AML relative to HDs, we individually compared *DNMT3A* ^WT^ and *DNMT3A* ^Mu^ patients with HDs and clarified their distinct profiles. Compared to HDs, it was *DNMT3A* ^WT^ but not *DNMT3A* ^Mu^-AML patients who showed a significant T-cell shift from the memory phenotype to Teff. In addition, *DNMT3A* ^Mu^-AML patients had a significantly decrease in CD8 Tn and an inverse distribution of Tcm and Tem within CD4 memory cells (Tcm increased and Tem decreased) compared with HDs. These results suggested that the existing of leukemia cells triggered T cells differentiation to the effector stage, but the defect in epigenetic regulation caused by *DNMT3A* mutations held the trend back. Considering that *DNMT3A* mutation was associated with poor outcomes in AML (24), its negative effect on the differentiation to Teff might be one mechanism.

We further explored the prognostic values of T-cell differentiation subsets’ proportions of AML, which has never been investigated in the large-scale cohort. As the major sub-population of both CD4 and CD8 T-cell populations, the prognostic significance of Tem in tumor has long been controversial. According to differentiation stages, Tem together with Teff belongs to late-differentiated cells, while according to cell types, it is classified into memory cells with a relatively longer survival time and stronger anti-tumor activity. In solid tumors, a higher proportion of Tem was reported to be associated with favorable clinical outcomes in triple-negative breast cancer as well as head and neck squamous cell carcinoma (30, 31), whereas Tiberti et al. found that it predicted poorer prognosis in colorectal tumors (32). In AML, an increased proportion of Tem was reported to be related to greater T-cell proliferative capacity and higher CR rates (7, 33), but another study showed that the proportion of Tem increased in relapsed AML (22). In the current study, by performing multivariate analysis including baseline characteristics and ELN-defined genetic risk category, we found that a lower proportion of CD4 Tem at diagnosis independently predicted poorer RFS in AML, which coincided with superior anti-tumor capacity of memory cells.

In addition, for the first time, we reported the prognostic significance of Tn in AML. Compared to the memory T cells, studies underlying the function and prognostic significance of naïve T cells were insufficient and inconsistent. A high absolute count of circulating CD4 Tn was an independent protective factor for progression-free survival in lung cancer (34). However, Takahashi et al. showed that CD8 Tn enrichment was an independent poor prognostic factor for both disease-free survival and overall survival in head and neck squamous cell carcinoma (30). In AML, CD8 Tn-derived donor cells effectively combated AML blasts in immunodeficient mice (35). Donor lymphocyte infusions (DLI) with cell products containing higher CD4 and CD8 Tn proportions were associated with a longer-term remission in AML (21). Consistently, our study demonstrated that increased BM CD8 Tn at diagnosis independently predicted both favorable RFS and EFS in AML. These findings indicated that CD8 Tn had a high anti-tumor potential and played a dominant role in the BM microenvironment despite of its relatively low proportions and limited direct anti-tumor effects in AML.

It is interesting to identify the correlation between the immunity-related factor with the leukemic cell-related indicator. In accordance with the prognostic significance, the low proportion of CD8 Tn was found to be associated with the adverse ELN genetic risk. Another consistency existed in *DNMT3A* ^Mu^ patients. *DNMT3A* mutations generally predicted poor outcome though not involved in ELN genetic risk factors (25). We found that *DNMT3A* ^Mu^ patients had decreased CD8 Tn and CD4 Tem than HDs, both of which were independent poor predictors for RFS. These results provide evidences for the view that interaction exists between leukemic cells and T cells, and both of them participated in the pathogenesis and progression of AML.

Age was found to affect the T-cell differentiation in the current study. Schnorfeil et al. reported that the storage of Tn was gradually depleted as aging in newly diagnosed AML (8). Xu et al. reported a decreased proportion of Tscm and an increased proportion of Tem as aging in healthy individuals, but not significant in AML cases (7). We found that the proportions of Tn and Tscm were negatively correlated with patient age, and those of Tcm and Tem were positively correlated with patients’ age in AML. HDs had the same trend with AML patients except for Teff. Considering the effect of age, it was important that AML patients had similar age distribution to HDs in the current study. Thus, the differences of the T-cell differentiation subsets’ proportions between AML patients and HDs were not attributed to age.

There were several limitations in the current study. First, this was a retrospective study, and the chemotherapy regimens for patients were not fully uniform. Even so, we found no statistical differences in patients’ survival among different induction regimens. Second, not all patients underwent NGS testing, and *DNMT3A* mutation screening was only covering partial patients of the entire cohort. Furthermore, *DNMT3A* mutations were not specifically detected in purified T cells. As a result, we grouped patients just according to the status of *DNMT3A* mutation testing using BM specimens.

## Conclusions

In conclusion, we undertook a large-scale cohort study to sketch the profile of BM T-cell differentiation subsets and clarify its prognostic significance in the newly diagnosed AML. Similar to HDs, AML patients displayed a distinct profile of differentiated T-cell sub-populations between CD4 and CD8 compartments. However, the distribution of T-cell differentiation subsets was different between AML patients HDs, and it was related to *DNMT3A* mutations. Compared to HDs, the T-cell compartment of *DNMT3A* ^WT^-AML patients skewed toward terminal differentiation at the expense of memory T cells, whereas *DNMT3A* ^Mu^-AML patients had a decrease in CD8 Tn and CD4 Tem as well as an increase in CD4 Tcm. The low proportions of CD4 Tem and CD8 Tn independently predicted poorer RFS and EFS. In addition, the low proportion of CD8 Tn was associated with adverse ELN genetic risk category. These results highlighted the separate and indispensable role of T cells in the pathogenesis and progression of AML, and provided support for more precise risk stratification and individualized immunotherapeutic selection for AML patients at diagnosis.

## Data availability statement

The raw data supporting the conclusions of this article will be made available by the authors, without undue reservation.

## Ethics statement

The studies involving humans were approved by the Ethics Committee of the Peking University People’s Hospital. The studies were conducted in accordance with the local legislation and institutional requirements. Written informed consent for participation was not required from the participants or the participants’ legal guardians/next of kin because this study used surplus samples after clinical testing, and the ethics approval is exempt from written information.

## Author contributions

KS: Data curation, Formal analysis, Investigation, Methodology, Validation, Writing – original draft. ZS: Writing – review & editing, Validation, Methodology, Formal analysis, Data curation. YW: Data curation, Methodology, Validation, Writing – review & editing. DX: Formal analysis, Validation, Writing – review & editing. YL: Formal analysis, Methodology, Validation, Writing – review & editing. QJ: Writing – review & editing, Validation, Investigation, Data curation. HJ: Writing – review & editing, Validation, Investigation, Data curation. XH: Data curation, Investigation, Supervision, Validation, Writing – review & editing. YQ: Conceptualization, Funding acquisition, Methodology, Project administration, Supervision, Validation, Writing – review & editing.
