# Supplementary material for: The profile and prognostic significance of bone marrow T-cell differentiation subsets in adult AML at diagnosis
Source: Front Immunol. 2024 Jul 19;15:1418792. doi: 10.3389/fimmu.2024.1418792 (PMC11294180; doi:10.3389/fimmu.2024.1418792)
Supplement: Supplementary file 1 [file DataSheet_1.docx]

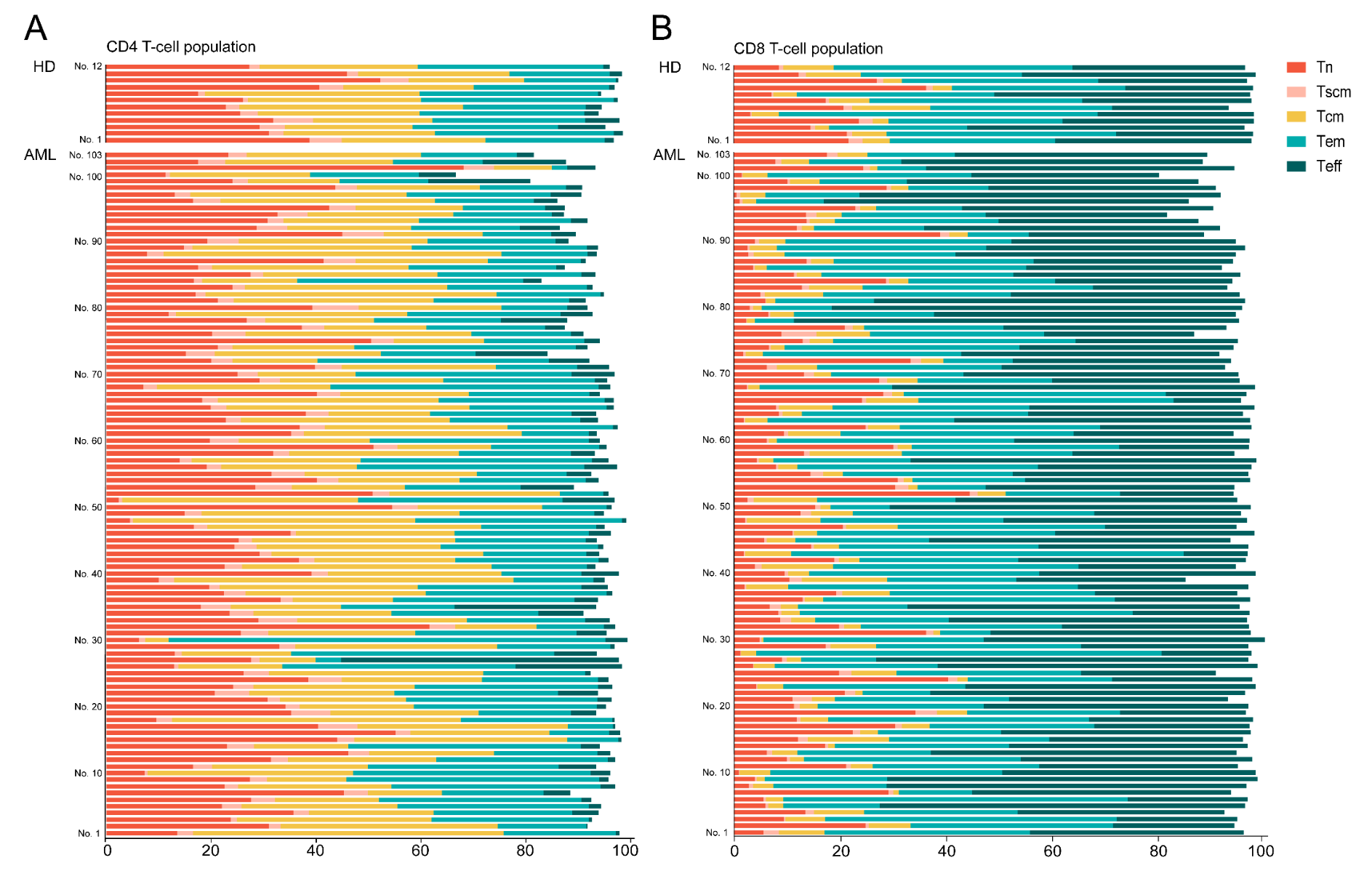
**Figure S1**. Distribution patterns of CD4 and CD8 T-cell differentiation subsets of the individual AML patients and HDs. CD4 T-cell differentiation subsets (A), CD8 T-cell differentiation subsets (B).

**Table S1**. Distribution of CD4 and CD8 T-cell differentiation subsets in AML patients and HDs. Represented by median (range).

| (%) | AML (n = 103) | | HD (n = 12) | *P* value |
| --- | --- | --- | --- | --- |
| CD4 T cells | |  |  |  |
| Tn | | 25.3 (2.5-68.2) | 30.3 (17.7-52.5) | 0.10 |
| Tscm | | 3.3 (0.5-8.8) | 3.1 (0.9-7.6) | 0.75 |
| Tcm | | 29.8 (4.5-64.8) | 28.9 (22.1-42.7) | 0.91 |
| Tem | | 24.4 (2.9-83.3) | 28.5 (17.5-36.8) | 0.29 |
| Teff | | 2.7 (0.2-53.0) | 1.7 (0.5-8.9) | 0.083 |
| Memory T cells | | 63.0 (17.1-93.9) | 62.6 (44.9-76.2) | 0.95 |
| CD8 T cells | |  |  |  |
| Tn | | 11.2 (0.6-44.7) | 19.1 (3.2-36.5) | 0.081 |
| Tscm | | 0.8 (0.06-6.7) | 1.1 (0.2—2.7) | 0.28 |
| Tcm | | 4.3 (0.5-17.5) | 5.1 (2.6-14.8) | 0.19 |
| Tem | | 32.3 (7.4-76.6) | 35.8 (26.2-60.1) | 0.18 |
| Teff | | 42.3 (12.1-84.1) | 32.3 (22.0-52.4) | 0.048 |
| Memory T cells | | 39.7 (9.0-83.1) | 42.2 (30.0-65.6) | 0.13 |

**Table S2**. The correlation between age and the proportions of CD4 and CD8 T-cell differentiation subsets in AML patients and HDs.

|  | AML (n = 103) | | HD (n = 12) | |
| --- | --- | --- | --- | --- |
| (%) | *r** | *P* value | *r** | *P* value |
| CD4 T cells |  |  |  |  |
| Tn | -0.21 | **0.031** | -0.23 | 0.48 |
| Tscm | -0.23 | **0.022** | -0.13 | 0.68 |
| Tcm | 0.27 | **0.0061** | 0.36 | 0.25 |
| Tem | 0.022 | 0.83 | 0.018 | 0.96 |
| Teff | -0.035 | 0.73 | -0.12 | 0.70 |
| CD8 T cells |  |  |  |  |
| Tn | -0.56 | **< 0.0010** | -0.25 | 0.43 |
| Tscm | -0.17 | **0.085** | 0.025 | 0.94 |
| Tcm | 0.30 | **0.0022** | 0.54 | **0.070** |
| Tem | 0.21 | **0.030** | 0.45 | 0.14 |
| Teff | 0.071 | 0.48 | -0.21 | 0.50 |

* Spearman *r* correlation coefficient was used.


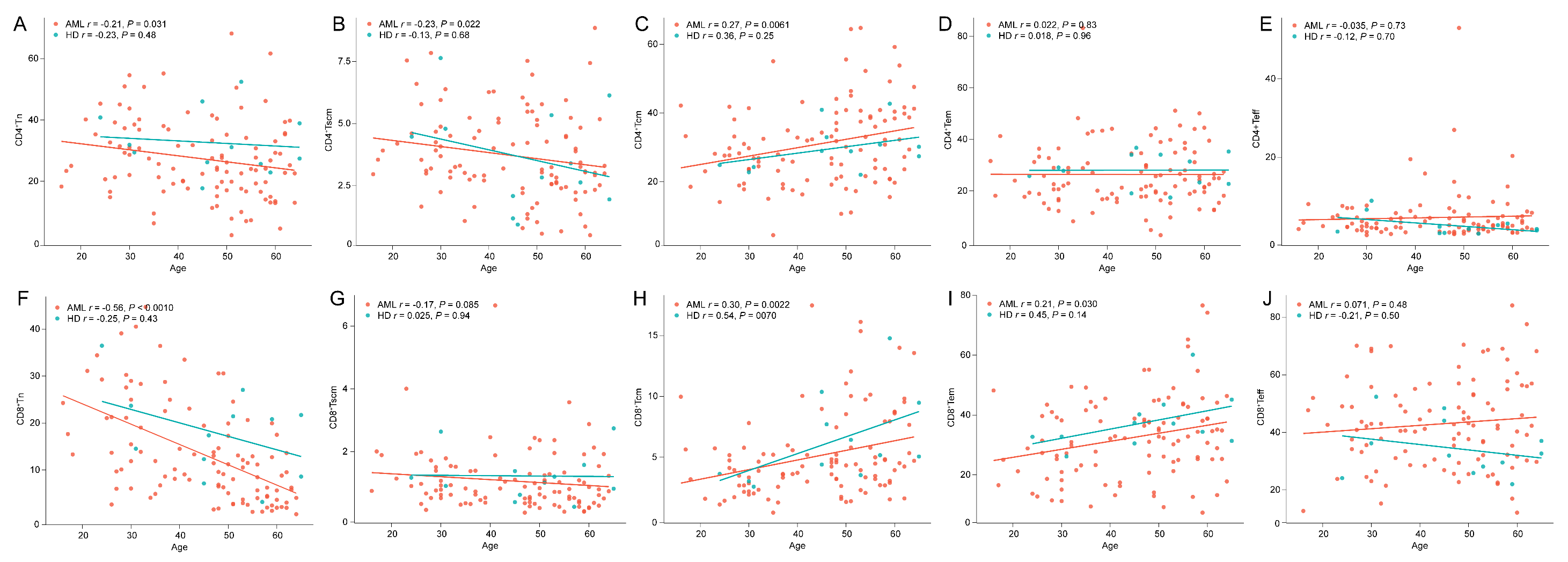


**Figure S2**. The correlation between age and the proportions of CD4 and CD8 T-cell differentiation subsets for CD4 Tn (A), CD4 Tscm (B), CD4 Tcm (C), CD4 Tem (D), CD4 Teff (E), CD8 Tn (F), CD8 Tscm (G), CD8 Tcm (H), CD8 Tem (I), andCD8 Teff (J) in AML patients and HDs. Correlation coefficient *r* was calculated using *Spearman* method.


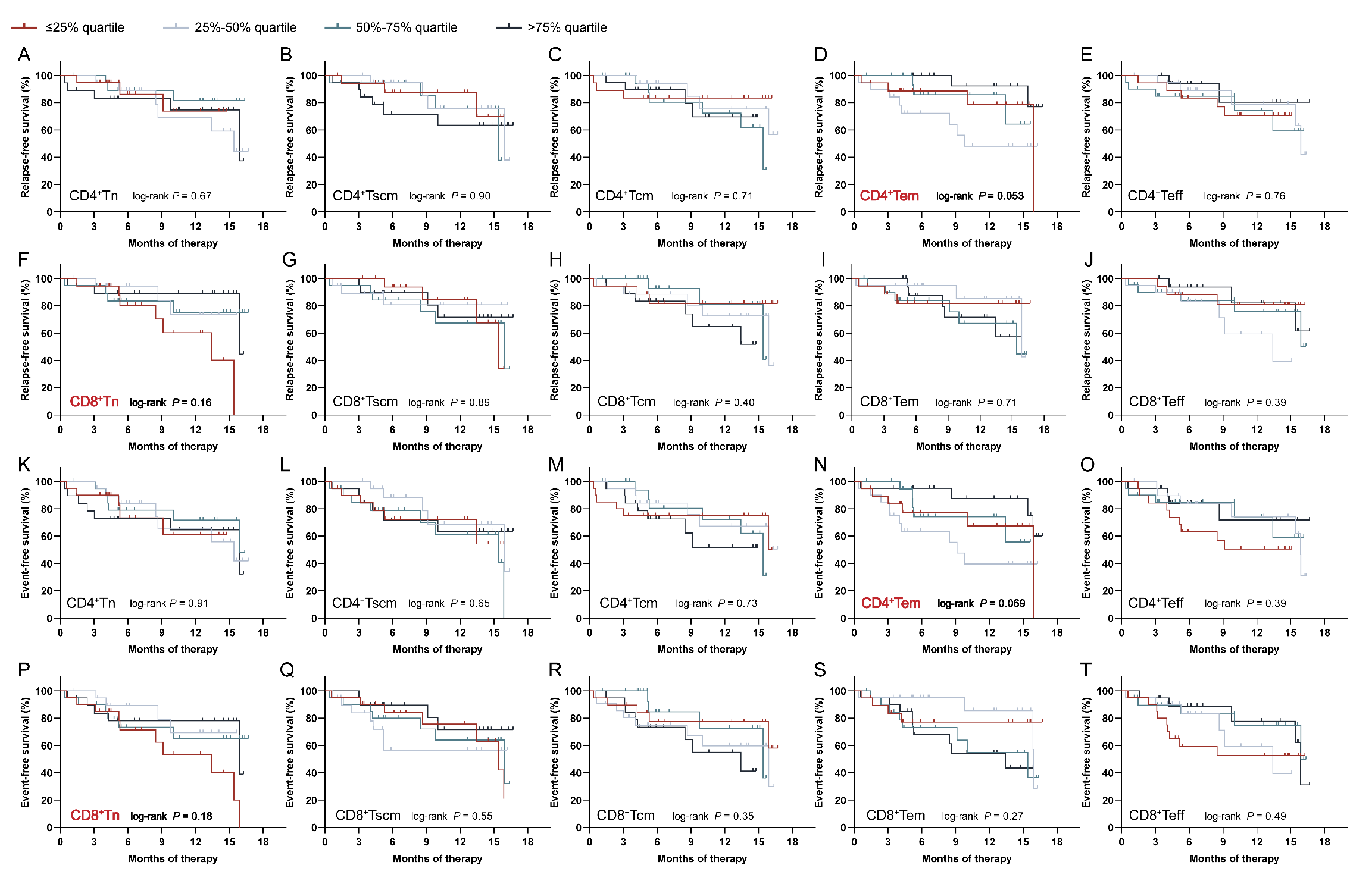


**Figure S3**. The impact of T-cell differentiation subsets’ proportions on RFS (A-J) and EFS (K-T) for CD4 Tn (A, K), CD4 Tscm (B, L), CD4 Tcm (C, M), CD4 Tem (D, N), CD4 Teff (E, O), CD8 Tn (F, P), CD8 Tscm (G, Q), CD8 Tcm (H, R), CD8 Tem (I, S), and CD8 Teff (J, T) grouped by quartiles.
